# Genetic and molecular alterations in olfactory neuroblastoma: implications for pathogenesis, prognosis and treatment

**DOI:** 10.18632/oncotarget.9683

**Published:** 2016-05-31

**Authors:** Piotr Czapiewski, Michał Kunc, Johannes Haybaeck

**Affiliations:** ^1^ Department of Pathomorphology, Medical University of Gdańsk, Gdańsk, Poland; ^2^ Faculty of Medicine, Medical University of Gdańsk, Gdańsk, Poland; ^3^ Department of Neuropathology, Institute of Pathology, Medical University of Graz, Graz, Austria

**Keywords:** olfactory neuroblastoma, esthesioneuroblastoma, mutation, comparative genomic hybridization, next generation sequencing

## Abstract

Olfactory neuroblastoma (ONB, Esthesioneuroblastoma) is an infrequent neoplasm of the head and neck area derived from olfactory neuroepithelium. Despite relatively good prognosis a subset of patients shows recurrence, progression and/or metastatic disease, which requires additional treatment. However, neither prognostic nor predictive factors are well specified. Thus, we performed a literature search for the currently available data on disturbances in molecular pathways, cytogenetic changes and results gained by next generation sequencing (NGS) approaches in ONB in order to gain an overview of genetic alterations which might be useful for treating patients with ONB. We present briefly ONB molecular pathogenesis and propose potential therapeutic targets and prognostic factors. Possible therapeutic targets in ONB include: receptor tyrosine kinases (c-kit, PDGFR-b, TrkB; EGFR); somatostatin receptor; FGF-FGFR1 signaling; Sonic hedgehog pathway; apoptosis-related pathways (Bcl-2, TRAIL) and neoangiogenesis (VEGF; KDR). Furthermore, we compare high- and low-grade ONB, and describe its frequent mimicker: sinonasal neuroendocrine carcinoma. ONB is often a therapeutic challenge, so our goal should be the implementation of acquired knowledge into clinical practice, especially at pretreated, recurrent and metastatic stages. Moreover, the multicenter molecular studies are needed to increase the amount of available data.

## GENERAL INFORMATION

Olfactory neuroblastoma (ONB) is a rare malignant tumor of the superior nasal cavity that was described for the first time in 1924. With a frequency of 0.4/million/year it accounts for approximately 2-3% of tumors of the nasal cavity [1]. Due to its occurrence mainly in the anatomical locations where olfactory epithelium normally exists as well as based on morphological features and expression of certain proteins characteristic of olfactory epithelium, ONB is believed to be derived from basal cells of olfactory epithelium [2, 3]. Tumors of very similar histology, with strict relationship to olfactory organs, were described among vertebrates, including fish, amphibians and mammals [4]. The Hyams’ histologic grading and Kadish staging system are the best studied factors correlating with prognosis, and thus useful for treatment planning. However, we lack specific guidelines for ONB treatment due to the limited data and the rarity of the disease. Craniofacial surgery, radiation therapy and chemotherapy are used in various combinations [5]. Surgical resection of ONB is usually combined with postoperative radiotherapy due to the high risk of locoregional recurrence [6]. Novel approaches include usage of endonasal endoscopy resection (EER), which allows for efficient local control and is associated with lower morbidity [7]. Chemotherapy as a neoadjuvant treatment is still under controversial debate and not recommended generally, however, one single study in a pediatric population reported promising results [8]. Other studies showed usefulness of adjuvant chemotherapy, especially in Kadish stage C high grade ONB [9]. Cisplatin-based chemotherapy, usually with etoposide, is an accepted mode of treatment in advanced, recurrent, especially high grade cases [10–13], but also non-platinum schemes (irinotecan, docetaxel, doxorubicin, ifosfamide, vincristine) were described to be effective [14]. Currently no studies are available showing correlation of expression of certain proteins regarding the response to chemotherapy. Similarly, little is known about specific therapeutic targets in ONB.

In this review article we report about main genetic disturbances, their correlation with prognosis, we describe concepts of molecular pathogenesis and potential targetable pathways in ONB. This should stimulate clinical pathologists, geneticists and oncologists to develop a grading system which better reflects the mutational state of the tumor and to investigate druggable targets for specific therapy.

## MOLECULAR PATHOGENESIS

The first molecular genetic analysis of ONB was performed by Carney et al. in 1995 [15]. The authors performed modified reverse-transcription polymerase chain reaction (RT-PCR) and found the ONB expressing *Drosophila achaete-scute gene* (*hASH1*), but not *Olfactory Marker Protein* (*OMP*) mRNA. *HASH1* is involved in immature olfactory neuronal development, whereas *OMP* is a marker of mature cells. This indicates ONB originates from immature neural crest cells of the olfactory epithelium. Additionally, hASH1 is responsible for neuroendocrine differentiation [16]. A recent study by Taggart et al. has confirmed ONB as well as other sinonasal neuroendocrine tumors (sinonasal neuroendocrine carcinoma, sinonasal undifferentiated carcinoma) as expressing hASH1 [17]. Its expression levels positively correlate with the grade of the tumor. In the diagnostic process, *hASH1* mRNA level evaluation may be used to distinguish sinonasal tumors with neuroendocrine differentiation from various poorly differentiated neoplasms of the sinonasal region like undifferentiated nasopharyngeal carcinoma, diffuse large B-cell lymphoma or malignant melanoma [18]. RNA interference studies revealed *hASH1* inhibition to lead to cell cycle arrest, so its overexpression may act as a trigger for cancer formation from olfactory epithelial cells [19]. Expression of hASH1 is down-regulated *via* the Notch dependent pathway [20]. Studies investigating this pathway in detail in ONB are missing, but its description might add a new puzzle stone for targeting this devastating tumor.

The moment at which a tumor acquires the ability to form new vessels as an effect of imbalance between pro-angiogenic and anti-angiogenic factors is called angiogenic switch, and neoangiogenesis is widely believed to promote tumor spread [21]. This process is only partially understood in ONB. STAT3 is activated by phosphorylation in ONB and subsequently increases the transcription of *HIF-1a* [22]. HIF-1a induces transcription of the erythropoietin (*Epo*) and erythropoietin receptor (*EpoR*) genes in ONB cells. These cells produce Epo, which acts in an autocrine manner and promotes neoangiogenesis. Bcl-2 acts as an anti-apoptotic factor but also contributes to the HIF-1a/Epo/EpoR/Bcl-2 system involved in angiogenesis [23]. hASH1 activates *BCL-2* transcription, so Bcl-2 inhibitors are promising candidates for treatment of high-grade ONB; also blocking of hASH1 can potentially block Bcl-2 activity [24]. In one single study Bcl-2 expression in ONB tended to be related with better response to neoadjuvant chemotherapy, but also with poorer prognosis [25]. Experimental data reported bortezomib to sensitize primary human ONB cells to TRAIL-induced apoptosis; combination of these agents effectively induced apoptosis in Bcl-2 positive primary tumor cells [26]. Moreover, another druggable protein and key player in new vessel formation, VEGF, is up-regulated *via* Bcl-2 in ONB cells [27]. In line bevacizumab, an anti-angiogenic agent stabilized the disease in a case of metastatic ONB for 28 months [28].

ONB cells express three main neurotrophin receptors: high affinity (TrkA, TrkB) and low affinity (p75NR) receptors. The first two are strongly expressed in almost all ONB cases, whereas p75NR is expressed in 60-100% [29]. Neurotrophins stimulate growth, differentiation and survival of neuronal cells. TrkB overexpression participates in tumorigenesis through ERK and Akt pathway activation. This enhances the maintenance of brain tumor-initiating cells (BTICs) and promotes lung adenocarcinoma metastasis formation [30, 31]. Similarly, p75NR promotes survival and proliferation of BTICs and this effect requires proper p75NR cleavage by α- and γ-secretases [32]. In turn, TrkA acts as proapoptotic and antiangiogenic factor and its expression is related to good prognosis in pediatric neuroblastoma (NB) [33]. Overexpression of TrkB in pediatric NB is associated with an unfavorable prognosis and resistance to chemotherapy. In a phase I clinical trial, the TRK inhibitor lestaurtinib has been shown to induce stabilization of disease in recurrent/refractory NB [34]. Combining conventional chemotherapy with more specific Trk inhibitors (e.g. entrectinib and GNF-4256) significantly inhibited NB growth in a xenograft mouse model [35]. Thus, efficacy of Trk inhibitors in ONB should be evaluated in future studies.

Mutant *TP53* is a driver of genetic alteration in various malignancies, like high-grade ovarian and colorectal carcinoma. Somatic *TP53* mutations occur in about 40,6% of head and neck cancers [36]. Contrary, mutations of *TP53* were not found in any of 19 cases of ONB, but about the half of all cases showed p53 overexpression [37]. Another study revealed p53 aberrant expression in 16 out of 26 cases (62%) [38]. Nevertheless, recent studies have identified point mutations of *TP53* in two cases of metastatic ONB [39, 40]. Aberrations of p53 probably appear at later stages during tumor development or progression and are not involved in the initial tumorigenesis.

## HIGH-GRADE VS. LOW-GRADE OLFACTORY NEUROBLASTOMA

ONB is graded according to the Hyams’ system, which despite its arbitrarity is widely used. ONB is dichotomically divided into low grade (Hyams’ I-II) and high grade (Hyams’ III-IV) [41]. However, a recent large study shows that division into low grade (Hyams’ I-III) and high grade cases (Hyams’ IV) reveals a better clinical correlation: high-grade ONB frequently presents with leptomeningeal metastasis, while low-grade ONB shows loco-regional recurrence [42]. With a median follow-up of 9.6 years, median disease free survival (DFS) and overall survival (OS) for resected low-grade ONB were 5.4 and 20.5 years, respectively. Conversely, median DFS and OS for high-grade ONB were 1.5 and 2.5 years, respectively. These controversies raise a question for the best clinical application of histopathological grading in clinics.

**Figure 1:**
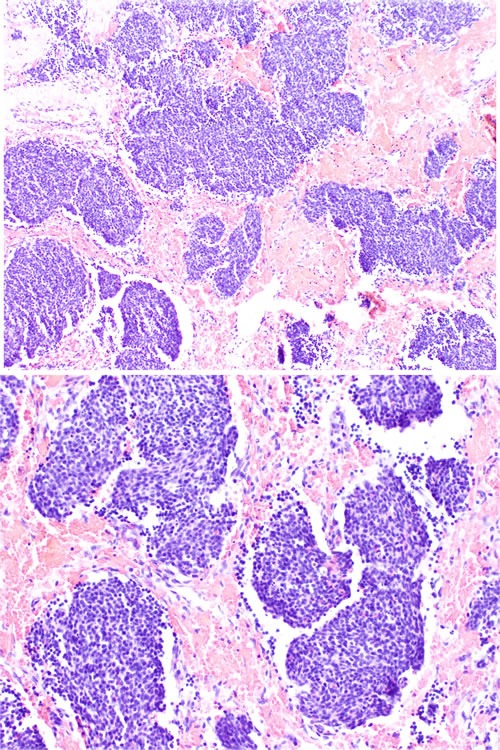
Low grade ONB Nests of small, monomorphic neoplastic cells with minimal cytological atypia.

**Figure 2:**
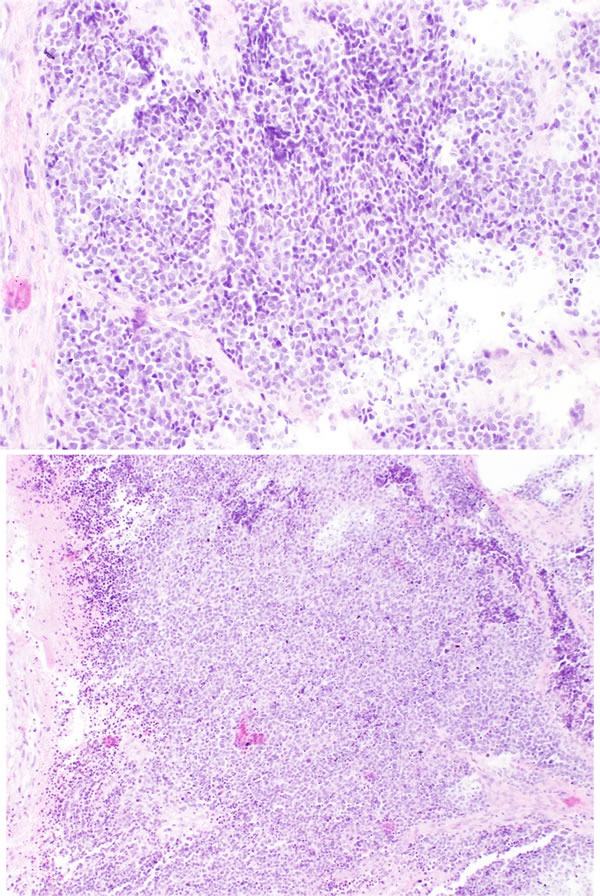
High grade ONB Neoplastic cells showing more diffuse pattern of growth. There is abvious cytological atypia of neoplastic cells.

Grade I tumors have a lobular architecture with the presence of a prominent neurofibrillary matrix. The cells are small and cytologically uniform without mitotic activity. Pseudorosettes (Homer-Wright rosettes) are often present and necrosis is not seen. Grade II tumors usually show less prominent matrix and more cellular atypia with mitoses. Grade III tumors may retain a lobular architecture but the cells are more atypical with increased mitoses and necrosis. True neural rosettes (Flexner-Wintersteiner) may be present. Grade IV tumors are the most undifferentiated and difficult to diagnose because there is often loss of lobular architecture. Cytologic atypia, necrosis, and mitotic activity are often present. Figure 1 and Figure 2 show, respectively, the histology of low and high grade ONB. However, in our opinion Hyams’ grading system may have some limitations. First, it was originally created based on an observation of small group of patients (only 17 from Armed Forced Institute of Pathology) [43]. Additionally, some patient's material used for confirmation of usefulness of this grade are taken from era before the identification of SNUC in 1990 and they might not have been reevaluated properly. It was shown that in non-specialized pathology department there is a huge overdiagnosis of small round cell tumors of sinonasal area as ONB. In only 2 of 12 tumors originally described as ONB the diagnosis confirmed in experienced Head and Neck Pathology Department [44].

There are ONB cases that do not fall easily into the Hyams’ Grading, for example if necrosis occurs in tumors that otherwise show features of Hyams’ Grade 1 or 2. It is not know which of these factors (lobular architecture preservation, mitotic index, nuclear pleomorphism, fibrillary matrix and rosettes) are most important for prognosis. Analysis of their reciprocal relationship has not been performed. Last but not least, despite multiple studies, there are no studies that clearly prove molecular differences between low- and high-grade ONB. These all facts underscore that Hyams’ system is certainly the only one accepted, but possibly not the best system for ONB grading.

## PROGNOSTIC AND PREDICTIVE FACTORS

Prognostic factors for ONB are not well characterized, mainly due to the rarity of these neoplasms and sparsity of studies with large case numbers. In the largest cohort described so far (124 cases) only high grade and age >65, but not stage, were associated with poor outcome [45]. Other studies revealed Kadish stage and lymph node metastases as correlated with poor prognosis [41]. The status of the resection margin was reported to positively correlate with survival [46]. One study points to a potential prognostic role of the mitotic index, presence of necrosis, spindle cell features and glandular hyperplasia, but this still remains to be confirmed in further studies [47]. At molecular level, high human telomerase reverse transcriptase (hTERT) immunoexpression is associated with metastasis occurrence and may be used as an ancillary prognostic marker [48]. Point mutations and wild type overexpression of *TP53* were observed in a subset of patients with recurrence or metastasis [37, 39, 40]. Weinreb et al. observed a statistically significant positive correlation between TrkA and GRP78 expression and OS [29]. The limitation of their study was uneventful disease course amongst sample as the majority of patients did not show any recurrence and a small cohort size. Longer survival may be associated with an increased abundance of S-100 protein-positive cells and a low Ki-67 labeling index [38], and this may probably be associated with differentiation, as the number of sustentacular cells decreases with increased tumor grade.

## CYTOGENETIC ALTERATIONS

Cytogenetic changes in ONB have been studied using different techniques, but are limited in number (main alterations are presented in Table 1). First approach to this issue was made by Hirose et al., who used flow cytometry [38]. These authors analyzed 22 cases of ONB and assessed their ploidy and percentage of cells in S phase. Most (43%) tumors were aneuploid, 22% diploid and 35% polyploid. Some diploid tumors exhibited ganglionic differentiation. The median percentage of cells in S-phase was 7.6%, but it varied from 1.5%-21.7%. However, no statistically significant survival difference was found for these variables. Most ONBs do not exhibit balanced translocations and the presence of fusion genes in patient samples and cell lines [49, 50]. Early research improperly suggested ONB exhibiting a translocation (11;22)(q24;q12) and producing an EWS/FLI1 transcript, which might cause misclassification to primitive neuroectodermal tumors (PNETs) [51, 52].

Bockmuhl and co-workers applied conventional comparative genomic hybridization (CGH) to 12 primary and 10 recurrent or metastatic ONBs [50]. This group determined frequent cytogenetic alterations and those associated with worse prognosis and metastases (Table 1). The study revealed ONBs showing high chromosomal instability and frequent loss of whole chromosomes or chromosomal arms. Specific deletion on chromosome 11 and gain on chromosome 1p were associated with metastasis and a worse prognosis. In up to 100% of cases deletions on chromosomes 3p and overrepresentations on 17q were observed.

Another study by Guled et al. with very strict morphological and immunohistochemical inclusion criteria involved 13 ONB cases [53]. Gains were more frequent than losses. High-stage tumors revealed more alterations (mean 28.5 changes per tumor) than low-stage ONB (mean 17 changes per tumor) and were more frequently aneuploidic. Among frequent changes gains of long arms of chromosomes 13 and 20 occurred in 50% of ONB, so were speculated to be particularly important for tumor progression.

Riazimand and coworkers in an analysis of 3 cases of ONB showed overrepresentations of chromosomal material of the entire chromosome 19, gains of some regions of the long arms of chromosomes 8, 15, and 22, and deletions of the entire long arm of chromosome 4 [54]. Beside these common aberrations, several single gains occurred on 6p, 10q, 1p, 9q, and 13q. A single analyzed case by Holland presented chromosomal aberrations predominantly involving chromosomes 2q, 5, 6q, 17, 19, 21q, and 22, as well as trisomy 8 [55], while another single case study (Szymas et al. [56]) showed gains of whole chromosomes 4, 8, 11, and 14, partial gains of 1q and 17q, partial deletions of 5q and 17q, and whole chromosome losses of 16, 18, 19, and X.

A recent study of 10 cases by Valli et al. argues that ONB has none recurrent chromosomal imbalances except gain of chromosome 19, which is consistent with Riazimand's findings [54], partial gains of the long arm of 20, deletions involving the long arm of 22 [57]. Only two cases exhibited multiple losses of entire chromosomes, while one case had no chromosomal alterations. Authors stated that neither Hyams’ grade nor Kadish stage was significantly correlated with any genetic alteration. Analysis of both, primary and relapsed tumor derived from one patient showed interesting differences between these two samples. Compared to primary tumor, relapsed ones had a reduced number of trisomies and an increased number of partial gains.

All these data indicate ONB as being characterized by high-level yet heterogeneous chromosomal instability (CIN). Paradoxically, high incidence of CIN may be responsible for a relatively indolent clinical course in most cases, since excessive CIN frequently hampers tumor progression [58]. Aneuploidic cells can be selectively targeted by energy and proteotoxic stress-inducing compounds (e.g. AICAR, 17-AAG and chloroquine), but their clinical usefulness in cancer therapy is yet to be determined [59]. Interestingly, platinum based agents are also thought to effectively kill cancer cells with CIN [60].

Several copy number changes identified in these studies were proposed to be associated with patient survival in other cancers entities. For example amplifications in the long arm of chromosome 20 have been associated with poor prognosis and chemotherapy resistance in breast and ovarian carcinoma [61, 62]. In head and neck squamous cell carcinoma 11q13 amplification is a marker of poor prognosis, whereas 3p and 11q loss is associated with resistance to chemotherapy and/or radiotherapy [63, 64]. Another frequent chromosomal aberration in ONB, 17q gain, characterizes pediatric NB with high risk of relapse [65]. Prospective studies should be performed to assess a possible role of these alterations in ONB.

**Table 1:** Most common chromosomal imbalances in ONB cases

Author	Clinical impact	DNA losses	DNA gains
Bockmühl [50]	Frequent alterations	1p21-31, 1q24-q32, 2q22-q32, 3p/q, 3p12-p14, 4p/q, 4p13-p15, 5p14, 5q, 6q14-q23, 9p, 9q22-q33, 10p/q, 10q26, 12p11.2-p12, 12q21, 13q, 13q21-q23, 18q, 21q21	1p34, 1q12, 1q23-q31, 7p21, 7q11.2, 7q31, 9p23-p24, 11q13, 14q, 14q32.2, 16p11.2, 16q, 16p13.3, 17p13, 17q21-q24, 17q12, 17q25, 17q11-q22, 19p/q, 20p, 20q, 20q13, 22q11.2, 22q13
	Worse prognosis	4p/q, 5p/q, 6q, 7q31-q32, 9p, 11p/q,15q21	1q12, 8q, 20q
	Metastases	5p/q, 6q, 7q31-q32, 11p12-p14, 11q14-q22, 15q21	1p32-p34, 1q12, 2p22-p24
Guled [53]	Frequent alterations	2q31.1, 2q33.3, 2q37.1, 3p21.31, 4p13, 5q31.2, 6p22.2, 6p21.33, 6p12.3, 6q16.3, 6q22.1, 15q11.2-q24.1, 15q13.1, 18q12.2-q12.3, 19q12, 19q13.11, 19q13.32, 19q13.43, 22q11.23, 22q12.1, 22q11.1-q11.21, Xp/q	1p36.31, 1p35.3, 4p16.2-p16.3, 4p12-p15.31, 4q12, 4q21.22-q22.1, 4q27-q35.2, 4q27-q35.2, 5q34, 5q35.1-q35.3, 6p12.3, 7q11.23, 7q21.11, 8q22-q24 9p13.3, 10p12.31, 12q23.1, 12q24.31, 13q, 13q14.2-14.3, 13q31.1, 13q34, 15q13.3, 16q12.1, 17q21, 20p/q, 20p13.3-p12.2, 20q11.21-q11.23, 20q13.32-q13.33, 21q, 22q12.1, Xp/q
	High-stage	2q31.1, 2q33.3, 6q16–q22, Xp21.1	5q35, 13q, 13q14.2–q14.3, 13q31.1, 20q11.21–q11.23
Riazimand [54]		4q, 6p	1p32, 8q24.1, 9q34.1, 10q24.3, 13q, 15q25, 19p/q, 22q
Holland [55] GTG banding		1p12- p21, 1p22-p32, 1p31-p33, 2q31-q33, 2q37, 3p11-p13, 3p12-p14, 3p25, 3q25, 3q26, 6p21, 6q12-q14, 6q22-q24, 10q26, 11q23, 15q26, 20q11.1-q12, 21q22, 22q13	1q25-q32, 1q25-q41, 16p13.3, 16q13-q22, 17p12, 17p13, 17q25
Holland [55] SNP array karyotyping		2q14.3, 3p21.3,3q27.2, 4p12, 4q31.3, 7q36.1, 8q24.3, 10p26.11, 11p11.2, 12q24.31, 14q32.33, 14q32.33, 16p11.2, 21q22.11	2q37.3, 3p21.3, 6q25.3, 6q27, 7q11.21, 7q11.23, 7q36.1, 7q36.3, 8p11.21, 9p13.3, 10q11.23, 11p14.1, 11q15.3, 11q23.3, 11q24.3, 13q12.11, 13.q33.3, 13q34, 14q32.31, 15q12, 15q13.1, 16p11.2, 16p13.11, 17q12, 17q21.31, 17q25.3, 19q13.42, 20q13.31, 22q13.31, 22q13.33
Szymas [56]		5q, 16p/q, 18p/q, 17p, 19p/q, Xp/q	1q, 4p/q, 8p/q, 11p/q, 14p/q, 17q
Valli [57]		1p31.1-p12, 1p33-q44, 3p/q, 8p/q, 10p/q, 21q11.2-q22.11, Yp11.31, Yp11.2, Yp11.21-q11.23	2p/q, 5p/q, 6p/q, 7p/q, 8p21.1-21.2 11p/q, 13p/q, 16p/q, 17p/q, 18p/q, 19p/q, 22p/q, Xp/q, Yp/q, 11q, 11q15.4-p15.5, 16p, 17p, 17q25.1-25.3, 20q13.2-q13.33, 21.q21.11-q22.3, 22q11.1-q13.31, 22q13.33

## GENOME SEQUENCING

Extensive sequencing of ONB is restricted to two cases of metastatic and one case of recurrent ONB. In the first study complex karyotypic disturbances led to the amplification of *FGFR1*, *FANCC*, *NOTCH1*, *CBFA2T3*, *RXRA*, *NSMAF*, *ASPH* as well as deletion of *JAZF1*, *ETS1*, *CCNH* and *F13A1* [39]. Sanger sequencing showed somatic nucleotide variants (SNVs) in the genes *MAP4K2*, *SIN3B*, *TAOK2*, *KDR*, *TP53*, *MYC*, *NLRC4* in metastatic tissue, but in the primary tumor, which had been resected many months before the primary tumor exhibited mutations only in *TP53*, *MAP4K2* and *TAOK2*. This highlights a possible role of mutations in *KDR*, *MYC*, *SIN3B*, *NLRC4* genes for the formation of metastases in ONB. Interesting interactions may occur between products of these genes: MYC attaches to SIN3B, while p53 to the NLRC4 promoter [66, 67]. In turn, whole exome sequencing of another metastatic ONB recognized 8 candidate cancer genes: *BRINP1*, *CARD11*, *CDKN2C*, *MEIS1*, *MINK1*, *PPP6C*, *TGFBR2* and *TP53* [40]. Their products may regulate cell cycle, responds to growth and differentiation factors or control apoptosis. Since mutations of both *TP53* and *CDKN2C* result in increased activity of CDK4/6, CDK4/6 inhibitors (palbociclib and LY2835219) were proposed as potential drugs in a patient [40]. Unfortunately, selected therapeutics have not yet been clinically introduced. Wang et al. have recently gone one step further: they identified mutated cancer-related genes with whole genome sequencing and subsequently implemented targeted therapy in a case of recurrent ONB [68]. Due to the mutations in the *EGFR*, *KDR*, *FGFR2*, *RET* genes they co-administered the EGFR-inhibitor cetuximab with the broad-spectrum tyrosine kinase inhibitor sunitinib to the patient. He achieved complete remission, which validates the clinical utility of genome-based precision medicine in ONB.

Some of the mutations mentioned above may be of potential predictive and prognostic utility. Cytotoxicity of cisplatin in pancreatic cancer cell lines was positively correlated with MAP4K2 expression, whereas NLRC4 deficiency may lead to radio-resistance of the respective tumor [69, 70]. Similarly p53 dysfunction results in resistance to radiotherapy and chemotherapy, including drugs widely used in ONB like etoposide [71]. Interestingly, only *TP53* mutations were found by both research groups performing genome sequencing of metastatic ONBs. In those two cases despite multimodal therapy including surgery, radiotherapy and chemotherapy the disease progressed. This may indicate that *TP53* mutation could be an unfavorable prognostic and predictive factor in ONB.

## POTENTIAL TARGETABLE PATHWAYS

Reports on usefulness of new targeted drugs in recurrent or metastatic ONB are restricted to case reports. Durable response was observed on therapy with sunitinib mesylate [68, 72], cetuximab [68], imatinib mesylate [73], bevacizumab [28] and temozolomide [74]. However, except in this one case, choice of these drugs was not supported by studies on possible genetic changes responsible for response. Nevertheless, expression of PDGFR-b and c-kit had been proven immunohistochemically in patients treated with sunitinib and imatinib, respectively [72, 73].

A subset of ONB expresses somatostatin receptors (SSTRs), thus the octreotide radionuclide scans have been used during the diagnostic process, including ACTH-secreting ONB [75]. A recent study on ONB with meningeal metastases has displayed overexpression of SSTRs, which was documented by PET/CT after the administration of a ^68^Gallium labeled somatostatin analog. Treatment with DOTA-D-Phe1-Tyr3-octreotid with the beta-emitter ^90^Yttrium (^90^Y-DOTATOC) reduced the size of the respective lesions [76]. Nevertheless, the therapeutic response to somatostatin analogs cannot be clearly predicted by the results of imaging tests [77]. Thus, immunohistochemistry for SSTRs may be indicated for proper future treatment planning.

Experimental studies demonstrated the Sonic hedgehog signaling pathway (Shh) being activated in ONB [78]. The influence of Shh signaling on the expression of its signaling components and cell cycle-related regulators was determined by immunoblotting and quantitative RT-PCR. This indicated treatment with cyclopamine to inhibit the proliferation and colony formation of ONB cells, to induce ONB cell cycle arrest and apoptosis, and to down-regulate the expression of *Patched1*, *Gli1* and *cyclin D1*. These data ask for using inhibitors of this pathway in pretreated, recurrent and metastatic disease. Usefulness of this *in vitro* study-based strategies has not yet been tested in clinical trials or case studies yet.

One study analyzed efficiency of inhibitors of IGF-1, PI3K/mTORC1/2, VEGFR, AKT/ERK, Shh and S6K1 in reducing viability of the ONB cell line TC268 [79]. The best obtained results involve combinations of AEW541 (IGF-1 inhibitor) and FS114 (S6K1 inhibitor) or sunitinib (VEGFR and other tyrosine kinases inhibitor) and FS115 (S6K1 inhibitor). Usage of BEZ235, which inhibits PI3K/mTORC1/2, had not been effective. This ineffectiveness was probably due to Akt/Erk phosphorylation and activation during treatment as their level increased upon treatment. Similar indicators of resistance mechanisms have been observed both in cell lines and in biopsies from cancer patients treated with inhibitors of relevant pathways [80, 81].

Weiss et al. reported *FGFR1* amplification in a case of metastatic ONB, which suggests FGFR1 inhibitors as potential therapeutic agents in a subset of ONB [39]. On the other hand, Schröck et al. used fluorescence *in situ* hybridization (FISH) to evaluate the *FGFR1* amplification status in sinonasal cancers, including seven ONB cases [82]. Contrary to some other tumors of sinonasal origin, especially squamous cell carcinoma, ONB showed wild type *FGFR1* copy number status. Interestingly, research conducted on ONB grown in athymic mice revealed that FGFR1 ligands, like bFGF, may induce tumor cell differentiation into olfactory supporting cell [83]. The olfactory epithelium of mice is composed of olfactory cells, progenitors of olfactory cells, these called globose basal cells, supporting cells, and horizontal basal cells [84]. Authors suggested bFGF-FGFR1 interaction to interrupt pathways supporting growth and survival of neuronal precursors, like BDNF-p75NR loop [85]. Additionally, bFGF was reported to exhibit cytotoxic effects on the ONB cell line JFEN. Similarly, TGF-alpha may induce tumor cells differentiation and even odor responsiveness, but without any cytotoxic effect [86]. These observations confirm that both bFGF and TGF-alpha intratumoral injections can potentially be active in ONB treatment by inducing differentiation of cancer cells.

Frequent amplifications of DNA at 20q in ONB may lead to transcription factor ZNF217 overexpression. In clear cell ovarian carcinoma ZNF217 overexpression was strongly related to poor prognosis upon platinum agent-based chemotherapy [61]. ZNF217 suppresses chemotherapy-induced cell death, promoting doxorubicin and paclitaxel resistance in breast cancer [87, 88]. Triciribine, a nucleoside analog and AKT inhibitor effectively kills chemoresistant cancer cells overexpressing ZNF217 [87]. As chemotherapy in ONB is often based on cisplatin, antracyclines and taxanes, triciribine may be potentially useful in overcoming resistance to these agents.

## OLFACTORY NEUROBLASTOMA VS. SINONASAL NEUROENDOCRINE CARCINOMA (SNEC)

In general, diagnosis of sinonasal tumors can be a challenging task as up to 30% of sinonasal malignancies referred to the Department of Pathology at The University of Texas MD Anderson Cancer Center are given a different diagnosis on expert review [89]. To be aware of all differential diagnoses is of important clinical relevance as prognosis for low grade ONB and SNEC and its mimicker SNUC (sinonasal undifferentiated carcinoma) varies significantly [45, 90–92]. However, high grade ONB, especially grade 4 can have a much worse prognosis (Czapiewski et al., Polish Journal of Pathology, in press) [6]. In particular in a group of 20 advanced (Kadish stage C) ONBs the respective 5-year and 10-year OS for low-grade ONB was 86% compared to 56% and 28% for high-grade ONB [93]. This poor prognosis of high grade cases could be, however, due to misdiagnosis of high grade sinonasal tumors, especially in not highly experienced centers. Location is also misleading in differential diagnosis as the majority of small cell SNEC develop in the superior or posterior nasal cavity, often extending into the maxillary or ethmoid sinuses [89].

One feature which should be investigated as a potential marker for the differential diagnosis of these tumors is the HPV status. HPV positivity has recently been described in SNEC in ¼ of cases [94], and the HPV status has not yet been evaluated in ONB. Additionally, HPV is almost always positive in oropharyngeal NEC, which may help in finding the right diagnosis in tumors involving the oropharynx [95]. Also sinonasal undifferentiated carcinoma (SNUC) can exhibit HPV positivity in between 6% to 47% of patients [96, 97].

Another challenging diagnosis is SNUC which especially in the past fell in the group of high grade ONB. The major problem when diagnosing SNUC can be some weak and/or focal neuroendocrine differentiation. One major open question still remains: is the neuroendocrine differentiation strong enough to report a diagnosis of ONB or SNEC. The major difference is the presence of cytokeratin reactivity in SNUC but no or weak and focal in ONB [89]. It is well known that the prognosis of SNUC can be similar to high grade ONB with an overall 5- and 10-year relative survival rate of 34.9% and 31.3%, respectively [98].

## SINONASAL NEUROENDOCRINE CARCINOMA: CLINICOPATHOLOGICAL FEATURES

As SNEC shares many similarities with ONB it is valuable to discuss some biological data on this infrequent sinonasal neoplasm. SNEC can exhibit morphological features resembling small cell carcinoma in the lung and extrapulmonary locations, it can also be similar to large cell neuroendocrine carcinoma.

Recently a large meta-analysis of 80 cases of sinonasal small cell carcinoma has been published [99]. This study has shown that 46.3% of patients were alive after a mean follow-up of 30.8 months, which is much higher result than for pulmonary small cell carcinoma. This study suggests that sinonasal small cell carcinoma may be less aggressive than pulmonary one. Therefore, direct extrapolation of treatment modalities from pulmonary to sinonasal small cell carcinoma may not be reasonable.

In a population based study on SNEC not divided into small cell and large cell neuroendocrine type the 5 year survival was similar (50,8%) to that of small cell type (46.3%) [100, 101]. In one large study from two central university hospitals the prognosis of head and neck SNEC was higher compared to gastrointestinal type tumors. On the other hand, disease specific survival differs substantially among locations and is 80.7%, 59.2%, 34.5%, and 33.0% for the sphenoid sinus, nasal cavity, maxillary sinus, and ethmoid sinus, respectively (p = 0.0014) [100].

Differences in survival among various anatomical location of SNEC are difficult to explain as we do not possess knowledge about disparities in molecular biology among them. Partially these dissimilarities may be secondary to more difficult therapeutic approach for example to the ethmoid sinus. Generalizing, DSS in ONB is much higher than in SNEC [100]. However, similarly to ONB, also in SNEC advanced stage disease (stages III to IV) is associated with poor survival outcomes compared to localized disease. Surgery with or without radio therapy in SNEC has lead to better results than radiotherapy alone [100]. For this reason one can take a result that surgery should be the first choice of treatment if the diagnosis SNEC or high grade ONB, is not certain based on the biopsy specimen.

In general, despite similar morphology and presence of neuroendocrine differentiation ONB and SNEC are biologically different neoplasms and so far there are no therapies than can be transmitted from neuroendocrine carcinomas, especially SNEC, to ONB treatment.

## FUTURE DIRECTIONS

Since ONB is an extremely rare entity, the multicenter molecular studies are needed to increase the amount of available data. It could help in finding correlation between the genetic landscape of ONB and its clinical course. Currently, we lack studies comparing genetic alterations of low-grade (I-III) and high-grade (IV) ONB and such studies should be conducted. By understanding the pathways that drive ONB with the help of cell lines studies and animal xenografts models, we might be able to target it. Special attention should be given to apoptosis-related molecules and pathways (e.g. Bcl-2, TRAIL); angiogenesis (VEGF, KDR); neurotrophin and somatostatin receptors and Shh pathway. Moreover, we need to establish the status of c-kit, PDGF-b and MGMT in ONB, since their inhibitors had showed clinical usefulness in single cases. Given its heterogeneity and rarity, we suggest that in selected cases of highly aggressive ONB, NGS sequencing and candidate cancer genes identification might be implemented. Such an approach may lead to discovery of completely new druggable driver genes in ONB.

## CONCLUSIONS

As we show in our article, there is a large data set on cell cycle and genetic alterations in ONB. Unfortunately, only few of these resources are routinely used in clinical practice, not even in a single case study. This review aims at highlighting future perspectives when exploring the genetic landscape of ONB. Our goal should be the implementation of acquired knowledge into clinical practice, especially at pretreated, recurrent and metastatic stages.
